# Clinical features and risk factors for outcomes of postoperative bacterial meningitis in pediatric neurosurgery: a retrospective study from 2013 to 2023

**DOI:** 10.3389/fped.2026.1749579

**Published:** 2026-05-15

**Authors:** Shuzhen Han, Qiaoxin Fang, Zhang Zhou, Xia Wu, Yingzi Ye, Lijing Ye, Jun Xu, Hui Yu

**Affiliations:** Department of Infectious Diseases, National Children’s Medical Center, Children’s Hospital of Fudan University, Shanghai, China

**Keywords:** bacterial meningitis, children, neurosurgery, pathogen, risk factors

## Abstract

**Objective:**

To explore clinical and etiological features and in-hospital mortality risk factors of post-operative bacterial meningitis in children who have undergone neurosurgery.

**Method:**

The main clinical manifestations were fever, disturbance of consciousness, and vomiting, with rare meningeal irritation signs. Gram-positive bacteria caused 53.4% of episodes and Gram-negative bacteria 46.6%, with the latter having a higher incidence of infections within 1 month postoperatively. The overall in-hospital mortality rate was 6.8%, and 85.7% of non-survivors had Gram-negative infections. Pulmonary infection, mechanical ventilation duration ≥7 days, and GCS score ≤8 were correlated with mortality (all *P* < 0.05).

**Results:**

A total of 99 children (103 infection episodes) were included. A retrospective study was conducted at Children's Hospital of Fudan University from 2013 to 2023. Clinical, etiological, and prognostic data were collected, with patients grouped by CSF culture results and prognosis. Differences between groups were analyzed using *t*-test, Wilcoxon rank-sum test, Chi-Square test, or Fisher's exact test. Kaplan–Meier survival curves and Log-rank test were used for survival analysis, and Cox regression identified factors associated with in-hospital mortality.

**Conclusions:**

Postoperative bacterial meningitis in children who have undergone neurosurgery has atypical clinical manifestations. Gram-positive bacteria are the main pathogens, but Gram-negative bacteria predominate in early postoperative infections (within 1 month), with Acinetobacter baumannii accounting for 45.8% and showing high carbapenem resistance. Pulmonary infection, mechanical ventilation duration ≥7 days, and GCS score ≤8 are associated with in-hospital mortality, necessitating early identification and intervention.

## 1 Introduction

Bacterial meningitis is a severe complication following neurosurgical procedures, which prolongs hospital stays, increases medical costs, and may even lead to severe neurological dysfunction and death in children. Previous reports have indicated that its incidence ranges from 0.5% to 21.4% (1–3), with a mortality rate of 2.76%–8% (4–6). Factors associated with an increased risk of post-neurosurgical meningitis include prolonged surgical duration, inadequate skin preparation of children, shunt infections, extended catheter placement time, and cerebrospinal fluid leakage (7–9).

Earlier studies suggested that Gram-positive bacteria were the main pathogens responsible for post-neurosurgical bacterial meningitis (7, 10). However, in recent years, some research has shown that Gram-negative bacteria have become the predominant pathogens (11–13), and meningitis caused by Gram-negative bacteria is associated with a higher mortality rate, even reaching up to 54.5% (13). With the increasing bacterial resistance, especially among Gram-negative bacteria, the failure rate of empirical treatment is also on the rise.

To better understand the clinical characteristics, pathogen distribution, antimicrobial susceptibility, and prognosis of post-neurosurgical bacterial meningitis in children, we analyzed children diagnosed with post-neurosurgical bacterial meningitis in our hospital from 2013 to 2023. This study aimed to explore their clinical features, identify risk factors affecting prognosis, and provide a basis for empirical treatment, thereby improving the survival rate and prognosis of affected children.

## 2 Method

### 2.1 Study population

From November 2013 to October 2023, children who underwent neurosurgery and had CSF tested in Children's Hospital of Fudan University were retrospectively evaluated for inclusion. Inclusion criteria: (1) Children with meningitis and positive CSF bacterial culture; (2) For conditional pathogens, ≥2 CSF cultures yielding the same bacteria; (3) Positive CSF culture from drainage tubes confirmed by the same bacteria in lumbar puncture CSF. Exclusion criteria: (1) Aseptic meningitis; (2) Tuberculous, fungal, viral meningitis, or mixed bacterial infections; (3) Patients/guardians refusing informed consent.

This study was approved by the Ethics Committee of Children's Hospital of Fudan University, and all subjects (or their parents) signed informed consent.

### 2.2 Data collection

Clinical data were collected via the hospital's medical record system, including clinical features, onset season, operation duration, time from surgery to infection, CSF routine and biochemical indicators before anti-infective treatment, bacterial characteristics, antimicrobial susceptibility, and prognosis. Data from children transferred postoperatively for further treatment were included, with initial clinical and laboratory data from the referring hospital used for analysis.

### 2.3 Group method

Patients were divided into Gram-positive and Gram-negative infection groups based on CSF culture, and into survival and non-survival groups based on discharge outcomes.

Diagnostic criteria for Post-Neurosurgical Bacterial Meningitis are as follows (7, 14): (1) Microorganisms cultured from CSF; (2) At least 2 symptoms (without other causes): ①Fever >38 ℃, headache, meningeal irritation, or cranial nerve involvement (age >1 year); ②Fever >38 ℃ or <36 ℃, apnea, bradycardia, or irritability (age ≤1 year); ③With at least one abnormal laboratory finding: CSF leukocytosis, elevated protein, reduced glucose; positive CSF Gram stain; positive blood culture; positive non-culture diagnostic tests of CSF, blood, or urine. Recurrence was defined as a second episode caused by a different pathogen or the same pathogen ≥3 weeks after completion of initial treatment (15).

### 2.4 Laboratory testing

Pathogen distribution and antimicrobial susceptibility testing (AST) were analyzed on these children. Pathogens were performed using matrix-assisted laser desorption ionization time-of-flight mass spectrometry (MALDI-TOF MS). Antimicrobial susceptibility was testing using the Vitek 2 Compact fully automated system. AST and its results were interpreted based on the Clinical and Laboratory Standards Institute guideline (2020 edition), with intermediate and resistant strains classified as non-susceptible (16). AST was performing for the following drugs: Penicillin, Erythromycin, Cefuroxime, Cefazolin, Ampicillin-Sulbactam, Oxacillin, Sulfamethoxazole (SMZ), Levofloxacin, Ciprofloxacin, Moxifloxacin, Nitrofurantoin, Gentamicin, Minocycline, Fosfomycin, Teicoplanin, Linezolid, Tigecycline, Vancomycin, Daptomycin, Dalfopristin, Rifampicin, Clindamycin, Tetracycline, Amikacin, Ceftazidime, Imipenem, Cefoperazone-Sulbactam, Cefepime, Piperacillin-Tazobactam, Meropenem, Piperacillin, Cefotaxime, Cefmetazole, Polymyxin, Tobramycin, Cefotetan and Ceftazidime-Avibactam.

### 2.5 Statistical analysis

For comparisons of continuous variables, Levene's test was first performed to assess variance homogeneity: the independent samples *t*-test was used for two groups with homogeneous variances, and the Welch-corrected *t*-test was applied for groups with heterogeneous variances; the Kruskal–Wallis H test was used for comparisons among three or more groups. Categorical variables were compared using the chi-square test, with Fisher's exact test employed when the expected frequency of any cell was less than 5. To account for multiple comparisons, the Benjamini–Hochberg (BH) method was utilized to adjust *P*-values and control the false discovery rate (FDR). Univariate survival analysis was conducted using Kaplan–Meier curves, and the log-rank test was used to compare survival differences between groups. Univariate and multivariate Cox proportional hazards regression models were constructed to explore the associations between various clinical factors and patient outcomes. All analyses were performed in SPSS software, version 25.0. A two-tailed *P* < 0.05 was considered statistically significant.

## 3 Result

### 3.1 General characteristics

From November 2013 to October 2023, a total of 616 children underwent neurosurgery and had CSF tested, with 128 cases of positive CSF culture (55 Gram-positive, 48 Gram-negative, 14 mixed Gram-positive, and 1 fungal) in Children's Hospital of Fudan University. Finally, 99 children (103 infection episodes) with confirmed bacterial meningitis and single pathogenic bacteria were included in our study. The reasons for the children's surgeries were as follows: 50 cases (48.5%) after brain tumor resection, 24 cases (23.3%) after hydrocephalus surgery, 11 cases (10.7%) after cerebral hemorrhage surgery, 11 cases (10.7%) after traumatic brain injury surgery, 6 cases (5.8%) after arachnoid cyst surgery, and 1 case (1.0%) after surgery for spina bifida with myelomeningocele.

The median interval from the primary neurosurgery to the clinical onset of BM was 21 days (IQR: 9–51 days). In early-onset cases (≤1 month post-surgery), the median onset was 11 days (IQR: 6–21 days), whereas in late-onset cases (>1 month post-surgery), the median onset was 71.5 days (IQR: 45.5–144.8 days). Pathogenic bacteria were definitively identified via culture and MALDI-TOF MS at a median of 23 days (IQR: 10–54 days) post-surgery, typically within 2–3 days of clinical symptom onset.

A total of 97 episodes (94.2%) of the children presented with fever, 57 episodes (55.3%) with disturbance of consciousness, 44 episodes (42.7%) with vomiting, 12 episodes (11.7%) with headache, 7 episodes (6.8%) with neck stiffness, 8 episodes (7.8%) with convulsions, and 8 episodes (7.8%) with abdominal pain. Basic information was showed in Supplementary Table 1.

### 3.2 Pathogen distribution

Among 103 episodes of children with post-neurosurgical bacterial meningitis, 55 episodes (53.4%) were caused by Gram-positive bacteria (Gram-positive infection group). Specifically, there were 49 episodes involving Staphylococcus species, including 35 episodes of Staphylococcus epidermidis, 7 episodes of Staphylococcus aureus [with 3 episodes being methicillin-resistant Staphylococcus aureus (MRSA)], and 7 episodes of other Staphylococcus species (5 episodes of Staphylococcus haemolyticus, 1 episode of Staphylococcus capitis, and 1 episode of coagulase-negative Staphylococcus). Additionally, there were 3 episodes of Enterococcus faecium, 1 episode of Enterococcus faecalis, 1 episode of viridans streptococci, and 1 episode of Streptococcus oralis.

The remaining 48 episodes (46.6%) were caused by Gram-negative bacteria (Gram-negative infection group). Among these, Acinetobacter baumannii accounted for 21 episodes (43.8%), followed by Klebsiella pneumoniae (8 episodes), Pseudomonas aeruginosa (5 episodes), Stenotrophomonas maltophilia (3 episodes), Serratia marcescens (3 episodes), Escherichia coli (2 episodes), and other Gram-negative bacilli (6 episodes). And the results of pathogen distribution were shown in Supplementary Table 2.

### 3.3 Antimicrobial susceptibility

In 103 episodes of children with post-neurosurgical bacterial meningitis, a total of 436 cerebrospinal fluid cultures were performed, yielding 277 bacterial strains. As shown in Table 1, all Gram-positive isolates (*n* = 55) were susceptible to vancomycin, linezolid, and tigecycline, with no resistant strains detected. Staphylococcus epidermidis (*n* = 35), the most prevalent Gram-positive bacterium, exhibited a high oxacillin resistance rate of 97.1% (34/35). For Staphylococcus aureus (*n* = 7), the oxacillin resistance rate was 3/7.

**Table 1:** Distribution of antimicrobial susceptibility of gram-positive Bacteria in cerebrospinal fluid from children with post-neurosurgical bacterial meningitis.

Characteristic	Gram-PositiveBacteria (*n* = 55)	Staphylococcus epidermidis (*n* = 35)	Staphylococcus aureus (*n* = 7)	MRSA (*n* = 3)	Staphylococcus haemolyticus (*n* = 5)	Enterococcus faecium (*n* = 3)	Enterococcus faecalis (*n* = 1)	Others (*n* = 4)
Penicillin	0	0	0	0	0	0	0	0
Erythromycin	7	7	0	0	0	0	0	0
Cefuroxime	5	0	4	0	0	0	0	1
Cefazolin	5	0	4	0	0	0	0	1
Ampicillin-Sulbactam	4	0	4	0	0	0	0	0
Oxacillin	6	1	4	0	0	0	0	1
SMZ	23	12	7	3	2	0	0	2
Levofloxacin	27	12	7	3	2	2	1	3
Ciprofloxacin	11	6	4	0	0	0	0	1
Moxifloxacin	12	7	4	0	0	0	0	1
Nitrofurantoin	15	7	4	0	0	2	1	1
Gentamicin	30	17	6	2	2	3	1	1
Minocycline	23	16	4	0	1	0	0	2
Fosfomycin	20	11	5	1	0	2	1	1
Teicoplanin	29	15	7	3	3	2	1	1
Linezolid	55	35	7	3	5	3	1	4
Tigecycline	55	35	7	3	5	5	1	4
Vancomycin	55	35	7	0	5	3	1	4
Daptomycin	0	10	4	2	4	0	0	1
Dalfopristin	0	0	0	0	0	0	0	2
Rifampicin	30	19	6	1	3	0	0	2
Clindamycin	5	4	0	0	0	0	0	1
Tetracycline	16	10	4	0	0	0	0	2

Among Gram-negative bacteria (Table 2), excluding Acinetobacter baumannii, the remaining isolates (*n* = 27) maintained relatively favorable susceptibility to quinolones (48.1%, 13/27), cefoperazone-sulbactam (44.4%, 12/27), and amikacin (59.3%, 16/27). However, their susceptibility to other antimicrobials was notably reduced: imipenem (48.1%, 13/27), piperacillin-tazobactam (37.0%, 10/27), and cefepime (14.8%, 4/27). Acinetobacter baumannii (*n* = 21) showed excellent susceptibility to polymyxin (100%, 21/21) and tigecycline (90.5%, 19/21), but lower susceptibility to meropenem (47.6%, 10/21), and only 28.6% (6/21) susceptibility to both amikacin and SMZ. Its susceptibility to other antimicrobial agents was even poorer. Klebsiella pneumoniae (*n* = 8) demonstrated relatively high susceptibility to tigecycline (5/8), SMZ (5/8), and amikacin (4/8), while its susceptibility to meropenem was low (2/8). Pseudomonas aeruginosa (*n* = 5) had a susceptibility rate of 3/5 to amikacin, ceftazidime, and cefoperazone-sulbactam, and 2/5 to meropenem, piperacillin-tazobactam, and cefepime, with poor susceptibility to other drugs. Stenotrophomonas maltophilia (*n* = 3) was fully susceptible to SMZ, levofloxacin, and minocycline (3/3), with 2/3 susceptibility to cefoperazone-sulbactam and only 1/3 susceptibility to ceftazidime.

**Table 2:** Distribution of antimicrobial susceptibility of gram-negative Bacteria in cerebrospinal fluid from children with post-neurosurgical bacterial meningitis.

Characteristic	Gram-NegativeBacteria (*n* = 48)	Acinetobacter baumannii (*n* = 21)	Klebsiella pneumoniae (*n* = 8)	Pseudomonas aeruginosa (*n* = 5)	Stenotrophomonas maltophilia (*n* = 3)	Serratia marcescen (*n* = 3)	Escherichia coli (*n* = 2)	Others (*n* = 6)
Amikacin	22	6	4	3	0	3	2	2
Ampicillin-Sulbactam	5	5	0	0	0	0	0	1
Ceftazidime	15	5	0	3	1	3	2	1
Imipenem	23	10	2	2	0	3	2	4
SMZ	19	6	5	0	3	3	0	2
Cefoperazone-Sulbactam	21	9	0	3	2	1	2	5
Cefepime	9	5	0	2	0	0	0	2
Piperacillin-Tazobactam	14	4	0	2	0	3	2	3
Meropenem	23	10	2	2	0	3	2	4
Tigecycline	25	19	5	0	0	0	0	1
Piperacillin	5	3	0	2	0	0	0	0
Cefazolin	0	0	0	0	0	0	0	0
Cefuroxime	0	0	0	0	0	0	0	0
Cefotaxime	2	2	0	0	0	0	0	0
Cefmetazole	2	2	0	0	0	0	0	0
Fosfomycin	7	3	3	0	0	1	0	0
Levofloxacin	21	8	2	1	3	3	0	4
Minocycline	20	14	1	0	3	0	0	2
Gentamicin	17	4	4	4	0	0	0	5
Ciprofloxacin	9	3	1	3	0	0	0	2
Polymyxin	0	21	0	1	0	0	0	0
Tobramycin	0	0	2	0	0	0	0	0
Cefotetan	0	0	1	0	0	0	0	0
Ceftazidime-Avibactam	0	0	2	0	0	0	0	0
Nitrofurantoin	0	0	0	0	0	0	1	0

### 3.4 Comparison between gram-positive and gram-negative infection groups

As shown in Table 3, Gram-negative group had higher rates of ICU stay (median 21 vs. 1 days, FDR-adjusted *P* = 0.009), pneumonia (68.8% vs. 41.8%, FDR-adjusted *P* = 0.028), and CSF protein levels (3,401.9 vs. 1,669.0 mg/L, *P* = 0.043). Gram-positive group showed higher GCS scores (12.55 ± 3.85 vs. 9.42 ± 4.08, *P* = 0.008) and CSF glucose levels (2.2 vs. 1.5 mmol/L, *P* = 0.012). No significant differences were found in gender, onset age, season, hospital stay, sepsis, mechanical ventilation, or surgical duration (all *P* > 0.05).

**Table 3:** Comparative characteristics of post-neurosurgical bacterial meningitis episodes between gram-positive and gram-negative bacterial infections.

Characteristic	Gram-positive episodes (*n* = 55)	Gram-negative episodes (*n* = 48)	*P*	*P’*
Sex, *n* (%)				
Male	37 (67.3)	30 (62.5)	0.612	0.640
Female	18 (32.7)	18 (37.5)		
Age at onset [yr, M (Q1, Q3)]	1.9 (0.8,5.0)	2.5 (1.6,5.0)	0.119	0.182
Season at onset, *n* (%)				
Spring	10 (18.2)	6 (12.5)	0.849	0.849
Summer	18 (32.7)	15 (21.3)		
Autumn	15 (27.3)	15 (31.3)		
Winter	12 (21.8)	12 (25.0)		
Clinical features				
Underlying condition				
Cancer	21 (38.2)	29 (60.4)	0.042	0.081
Hydrocephalus	17 (30.9)	7 (14.6)	0.051	0.084
ICU admission				
ICU stay [d, M (Q1,Q3)]	1.0 (0,14.0)	21 (4.3,46.3)	<0.001	0.008
Pneumonia	23 (41.8)	33 (68.8)	0.006	0.028
Sepsis	17 (30.9)	21 (43.8)	0.178	0.256
Mechanical ventilation ≥24 h	37 (55.2)	30 (44.8)	0.612	0.640
GCS score	12.55 ± 3.85	9.42 ± 4.08	<0.001	0.008
Surgical parameters				
Surgical duration ≥4 h	13 (44.8)	16 (55.2)	0.275	0.333
V-P shunt	28 (50.9)	9 (18.8)	<0.001	0.008
External drainage	23 (41.8)	26 (54.2)	0.211	0.270
Ommaya reservoir	39 (70.9)	23 (47.9)	0.017	0.043
Catheter-related infection	17 (30.9)	5 (10.4)	0.011	0.042
CSF leak/Surgical site infection	12 (21.8)	21 (43.8)	0.017	0.043
Laboratory findings				
Blood tests				
WBC × 10^9^/L	15.25 ± 8.10	12.45 ± 5.90	0.051	0.084
CRP≥8 mg/L, *n* (%)	36 (65.5)	37 (77.1)	0.195	0.264
Cerebrospinal fluid				
WBC × 10^9^/L	300 (70,550)	385 (129.5, 1,392.5)	0.036	0.075
Glucose (mmol/L) [M (Q1,Q3)]	2.2 (1.2,2.7)	1.5 (0.1,2.2)	0.002	0.012
Protein (mg/L) [M (Q1,Q3)]	1,669.0 (842.0, 3,404.0)	3,401.99 (1,419.2, 4,864.1)	0.016	0.043
Outcomes				
Hospital stay [d, M (Q1,Q3)]	52.0 (30.0,69.0)	53.0 (27.3,95.3)	0.433	0.498
Postoperative onset <30 d	30 (45.5)	36 (54.5)	0.031	0.071

GCS, Glasgow Coma Scale; CSF, cerebrospinal fluid; WBC, White blood cell; CRP, C-reactive protein.

*P*’ FDR adjusted *P* value.

### 3.5 Survival analysis

Seven deaths occurred (6.8%), 6 caused by Gram-negative bacteria (85.7%). The median time from surgery to death was 38 days (range: 27–219 days), and the median time from BM diagnosis to death was 18 days (range: 0–151 days). The direct causes of mortality in these patients included central respiratory failure, brain herniation, and sepsis complicated by multiple organ dysfunction syndrome. Non-survival group showed lower GCS scores (4.00 ± 2.16 vs. 11.60 ± 3.88, FDR-adjusted *P* = 0.008) and mechanical ventilation ≥7 days (100% vs. 27.1%, *P* = 0.008) (Table 4).

**Table 4:** Comparative characteristics of post-neurosurgical bacterial meningitis episodes by survival Status.

Characteristic	Survivors (*n* = 96)	Non-survivors (*n* = 7)	*P*	*P’*
Sex, *n* (%)				
Male	64 (66.7)	3 (42.9)	0.387	0.470
Female	32 (33.3)	4 (57.1)		
Age at onset [yr, M (Q1, Q3)]	2.0 (1.0,4.8)	4.0 (1.8,9.0)	0.178	0.304
Season at onset, *n* (%)				
Spring	15 (15.6)	1 (14.2)	0.243	0.373
Summer	30 (31.3)	3 (42.9)		
Autumn	30 (31.2)	0 (0)		
Winter	21 (21.8)	3 (42.9)		
Clinical features				
Underlying condition				
Cancer	45 (46.9)	5 (71.4)	0.388	0.470
Hydrocephalus	24 (25.0)	0 (0)	0.295	0.424
ICU admission	13 (13.5)	3 (42.9)	0.015	0.053
Length of ICU stay [d, M (Q1, Q3)]	9.5 (0, 29.8)	38 (10.0, 87.0)	0.015	0.053
Pneumonia	49 (51.0)	7 (100.0)	0.034	0.098
Sepsis	33 (34.4)	5 (71.4)	0.120	0.230
Mechanical ventilation ≥7 days	26 (27.1)	7 (100.0)	<0.001	0.008
GCS score [mean + SD]	11.6 ± 3.9	4.0 ± 2.2	<0.001	0.008
GCS score ≤ 8	17 (17.7)	6 (85.7)	<0.001	0.008
GCS score >8	79 (82.3)	1 (14.3)		
Surgical parameters				
Surgical duration ≥4 h	27 (28.1)	2 (28.6)	1.000	1.000
VP shunt	37 (38.5)	0 (0)	0.100	0.209
Ommaya reservoir	61 (63.5)	1 (14.3)	0.003	0.017
Catheter-related infection	21 (21.9)	0 (0)	0.368	0.470
CSF leak/Surgical site infection	35 (36.5)	3 (42.9)	1.000	1.000
Laboratory findings				
Gram-nagative infection	42 (43.8)	6 (85.7)	0.048	0.123
Blood tests				
WBC × 10^9^/L	14.1 ± 7.2	12.4 ± 8.0	0.574	0.660
CRP≥8 mg/L, *n* (%)	66 (68.8)	7 (100)	0.185	0.304
Cerebrospinal fluid, [M (Q1,Q3)]				
WBC × 10^6^/L	335.0 (87.5, 987.5)	300.0 (70.0, 1,100.0)	0.758	0.830
Glucose (mmol/L)	2.0 (0.6, 2.4)	0.1 (0.1, 1.9)	0.016	0.053
Protein (mg/L)	2,053.5 (1,018.0, 3,813.4)	3,816.0 (1,814.0, 5,896.1)	0.056	0.129

GCS, Glasgow Coma Scale; CSF, cerebrospinal fluid; WBC, White blood cell; CRP, C-reactive protein.

*P*’ FDR adjusted *P* value.

**Figure 1:**
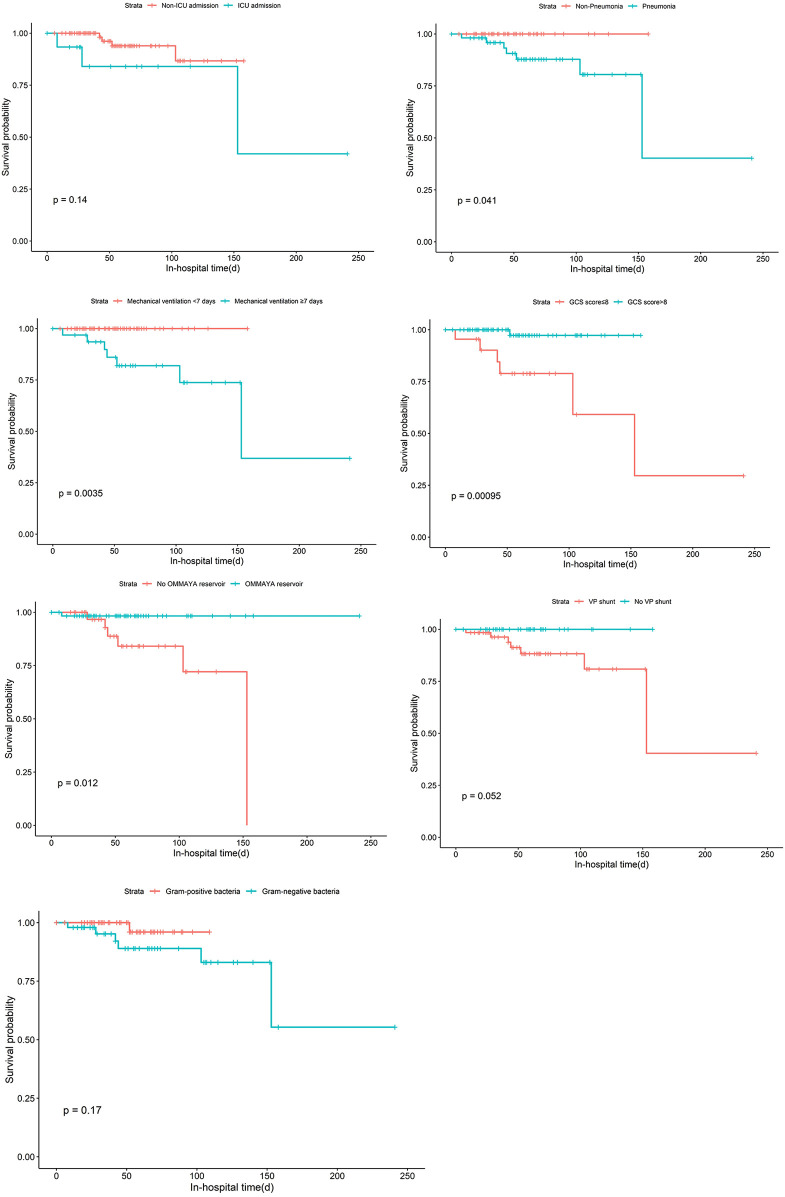
Kaplan–Meier curves of in-hospital mortality in pediatric post-neurosurgical bacterial meningitis stratified by clinical variables.

Kaplan–Meier curves were constructed to evaluate the in-hospital mortality of children with PNM, stratified by different clinical factors (Figure 1). For the stratification of ICU admission status, the survival probability showed a trend, but the difference was not statistically significant (*P* = 0.14). When stratified by the presence of pneumonia, children with pneumonia had a significantly lower survival probability compared to those without pneumonia (*P* = 0.041). Mechanical ventilation duration also had a notable impact: children requiring mechanical ventilation for ≥7 days had a much lower survival probability (*P* = 0.0035). The GCS score stratification revealed that a lower GCS score (GCS≤8) was associated with a drastically reduced survival probability (*P* = 0.00005). Children with OMMAYA reservoir had a significantly lower survival probability (*P* = 0.012). The presence of a VP shunt also affected survival, although the difference was of marginal significance (*P* = 0.052). Finally, stratification by bacterial type (Gram-positive vs. Gram-negative bacteria) showed no significant difference in survival probability (*P* = 0.17).

Unvariate Cox proportional hazards regression was performed to assess the association of clinical factors with outcomes of postoperative bacterial meningitis in pediatric neurosurgery. Variables with statistical significance were further included in multivariate analysis. Compared to children without pneumonia, those with pneumonia had an unadjusted hazard ratio (HR) of 43.24 (95% CI: 0.07–26,296.18), but this was excluded from the multivariate model due to non-significant statistical evidence. Children requiring mechanical ventilation for ≥7 days (vs. <7 days, reference) showed an unadjusted HR of 93.07 (95% CI: 0.18–48,669.94, *P* = 0.156) and were also excluded from multivariate analysis. A GCS score >8 (vs. ≤8, reference) was associated with a significantly reduced hazard in both univariate (HR = 0.06, 95% CI: 0.01–0.53, *P* = 0.011) and multivariate (adjusted HR = 0.09, 95% CI: 0.01–0.78, *P* = 0.028) analyses, indicating that a higher GCS score is an independent protective factor. Additionally, use of an OMMAYA reservoir (vs. non-use, reference) was associated with a reduced hazard in univariate analysis (HR = 0.10, 95% CI: 0.01–0.86, *P* = 0.035), though this association lost statistical significance in the multivariate model (adjusted HR = 0.17, 95% CI: 0.02–1.43, *P* = 0.103). The result was shown in Table 5.

**Table 5:** Multivariate Cox proportional hazards regression analysis of independent hazard ratios for outcomes of postoperative bacterial meningitis in pediatric neurosurgery.

Characteristics	Unadjusted HR (95%CI)	*P*	Adjusted HR (95% CI)	*P*
Pheumonia				
Non-Pheumonia	Reference		Reference	
Pheumonia	43.24 (0.07, 26,296.18)	0.249	—	—
Mechanical ventilation				
<7 days	Reference		Reference	
≥7 days	93.07 (0.18, 48,669.94)	0.156	—	—
GCS score				
≤8	Reference		Reference	
>8	0.06 (0.01, 0.53)	0.011	0.09 (0.01, 0.78)	0.028
OMMAYA reservoir				
No-user	Reference		Reference	
User	0.10 (0.01, 0.86)	0.035	0.17 (0.02, 1.43)	0.103

## 4 Discussion

Post-neurosurgical meningitis typically presents with atypical clinical manifestations, predominantly characterized by fever, which may be accompanied by varying degrees of altered mental status, sepsis, or peritonitis. Meningeal irritation signs are relatively infrequent (7, 17). In the present study, fever (94.2%) was the most common clinical feature, followed by altered mental status (55.3%) and vomiting (42.7%). Headache was less prominent (11.7%), potentially attributable to the young age of the pediatric cohort—with limited verbal expression abilities—and the presence of varying degrees of consciousness disturbance in some cases. Only 6.8% of children exhibited nuchal rigidity. Clinicians should therefore maintain a high index of suspicion for meningitis in the presence of temperature fluctuations, systemic discomfort (e.g., headache, vomiting, abdominal pain, seizures), or altered mental status. Concurrently, targeted parental education regarding post-neurosurgical monitoring is imperative to facilitate early recognition of infectious signs, thereby enabling prompt diagnosis, timely intervention, and improved prognostic outcomes.

While no significant seasonal variation in the incidence of post-neurosurgical meningitis was observed in this study, cases were more prevalent during summer and autumn, consistent with findings reported by Luo et al. (18) from Shanghai Children's Medical Center. This trend may be linked to the humid and hot climatic conditions in southern China during these seasons, which favor bacterial proliferation, coupled with increased susceptibility to cutaneous and gastrointestinal infections in children. Post-neurosurgical meningitis may also arise from catheter colonization during surgery, craniotomy incision infections, retrograde infection via shunt catheters secondary to cerebrospinal fluid leakage, or bacterial invasion of the central nervous system from adjacent wound sites (7, 17). Enhanced postoperative wound care and proactive management of gastrointestinal diseases—particularly during summer and autumn—may thus mitigate the risk of meningitis in post-neurosurgical pediatric patients in southern regions.

The pathogenic spectrum of post-neurosurgical meningitis remains debated: while most reports identify Gram-positive bacteria as the primary etiologic agents (7, 10), others highlight Gram-negative predominance (11, 12). In the current study, Gram-positive bacteria accounted for 53.4% of cases overall; however, Gram-negative pathogens predominated in meningitis occurring within 1 month of surgery (54.5%), whereas Gram-positive bacteria were more common in infections developing ≥1 month postoperatively (67.6%)—aligning with the observations of Luo et al. (18) from Shanghai Children's Medical Center and Kurtaran et al. from All India Institute of Medical Sciences (12) similarly noted that Gram-positive meningitis occurs significantly later than Gram-negative meningitis and is more frequently associated with shunt procedures. The high prevalence of Gram-positive infections in our cohort may be related to the fact that 83.5% of patients had ventriculoperitoneal or external drainage, and 60.2% had OMMAYA reservoirs or subcutaneous adjustable pressure valves implanted.

Vancomycin in combination with β-lactam antibiotics is recommended as first-line empirical therapy for healthcare-associated ventriculitis and meningitis7. In this study, no Gram-positive isolates resistant to vancomycin, linezolid, or tigecycline were detected. Notably, the in-hospital mortality rate in the Gram-positive infection group (1.8%, 1/55) was significantly lower than that in the Gram-negative group (12.5%, 7/48), potentially reflecting the efficacy of early empirical vancomycin use. The rising incidence of Acinetobacter baumannii meningitis, coupled with increasing carbapenem resistance (19)—with reported resistance rates of 40% 18% and 70% (20) in CSF isolates—poses a critical challenge. Current guidelines advocate for colistin or polymyxin B as alternatives to meropenem in such cases, often administered via intraventricular or intrathecal routes (7, 19, 21). In our cohort, A. baumannii exhibited a carbapenem resistance rate of 52.4% (11/21) but 100% susceptibility to polymyxin. Among 5 patients treated with combined intravenous and intrathecal polymyxin, 4 showed clinical improvement, while 1 succumbed to multi-organ failure after 8 months of hospitalization.

The accurate capture of this complex pathogen spectrum in our study was significantly bolstered by the clinical application of MALDI-TOF MS. As emphasized in the IDSA guidelines for healthcare-associated ventriculitis and meningitis (7), the clinical presentation of these infections—especially in pediatric and neurosurgical populations—is often variable and non-specific, making precise laboratory identification paramount. While the guidelines acknowledge both staphylococci and resistant Gram-negative bacilli as typical etiologic agents, our findings reveal a significant regional burden of carbapenem-resistant A. baumannii. Specifically, A. baumannii accounted for nearly half of all Gram-negative infections in our cohort, with a carbapenem resistance rate exceeding 50% (11/21). This highlights a critical therapeutic challenge that may be more pronounced in our setting compared to the epidemiology described in some Western clinical reports. These distinct regional characteristics further underscore the necessity of high-resolution diagnostic modalities like mass spectrometry to guide targeted antimicrobial therapy.

Adult studies report mortality rates of 15%–35% for post-neurosurgical meningitis/encephalitis, with risk factors including male sex, CSF leakage, comorbidities, early reoperation, prolonged surgery, external ventricular drainage (EVD), lumbar drainage (LD), and diabetes (22–25). Sharma et al. (19) identified age >40 years, GCS score ≤8, presence of EVD, CSF leukocyte count >200 cells/mm^3^, and comorbidities as predictors of mortality in Acinetobacter meningitis/ventriculitis. However, data on prognostic factors in pediatric post-neurosurgical meningitis remain scarce. This study is the first to analyze mortality-associated factors in this population, and we found that key factors for death included ICU admission, pneumonia, mechanical ventilation ≥7 days, GCS score ≤8, and Non-OMMAYA reservoirs—with particularly poor survival in children. The Glasgow Coma Scale is widely used as a predictive indicator for patients with head injuries due to its ease of application, simplicity, and rapidity (26, 27). A study by Shi et al. (28) demonstrated that a GCS score ≤8 is an independent risk factor for mortality in patients with post-neurosurgical Enterobacteriaceae meningitis/encephalitis. In the present study, there was a statistically significant difference in GCS scores between non-survivors and survivors. These findings indicate that children with lower GCS scores are more likely to have poor prognoses, reminding clinicians to formulate effective antibiotic regimens early to reduce mortality. Compared with traditional EVD and ventriculoperitoneal (V-P) shunt, Ommaya reservoirs have the advantages of smaller surgical trauma, shorter operation time, lower risk of retrograde infection, and the ability to administer drugs intra-cystically, thus being widely used in pediatric neurosurgical procedures (29). In our study, it was found that children with an OMMAYA reservoir implantation may have a better prognosis. However, it may require validation with a larger sample size.

This study is a single-center research with a relatively small total number of cases, and the number of deaths is also small. The subjects excluded children with aseptic meningitis, mixed infections, as well as those with viral, fungal, or tuberculous meningitis, including only children with a single positive bacterial culture in cerebrospinal fluid. However, the positive rate of CSF culture is low, especially in those who have received antimicrobial agents before CSF culture, where the positive rate of CSF culture will drop to an even lower level. Therefore, there exists incompleteness in the data, lacking universal applicability. To better illustrate the associations between potential influencing factors and the outcomes of these pediatric patients, a multivariate Cox proportional hazards regression analysis was performed. However, due to the small number of death events, the resulting confidence intervals were excessively wide, and overfitting may have occurred. Therefore, the conclusions derived from this model should be interpreted with caution. Finally, in our study, apart from routine blood tests and CRP, no other laboratory indicators (such as PCT, IL-6, ferritin, D-dimer, blood lactic acid, etc.) were included. In the future, it is necessary to further expand the sample and conduct multi-center studies to more accurately reflect the clinical characteristics of post-neurosurgical meningitis in children.

## Data Availability

The datasets used and/or analyzed during the current study are available from the corresponding author on reasonable request.
