# Supplementary material for: Clinical features and risk factors for outcomes of postoperative bacterial meningitis in pediatric neurosurgery: a retrospective study from 2013 to 2023
Source: Front Pediatr. 2026 May 15;14:1749579. doi: 10.3389/fped.2026.1749579 (PMC13219268; doi:10.3389/fped.2026.1749579)
Supplement: Supplementary file 1 [file Table1.docx]

**Supplementary Table 1. Basic information of Microorganisms Isolated from Cerebrospinal Fluids of Post-Neurosurgical Bacterial Meningitis Children.**

| **Characteristic** | **All episodes**  **(n=103)** |
| --- | --- |
| **Sex, the number of cases (%)** |  |
| Male | 65 (65.7) |
| Female | 34 (34.3) |
| **Age at onset [yr, M(Q1, Q3)]** | 1.92 (0.83, 5.00) |
| **Type of primary surgery** |  |
| Brain tumor resection | 50 (48.5) |
| Hydrocephalus surgery | 24 (23.3) |
| Cerebral hemorrhage surgery | 11 (10.7) |
| Traumatic brain injury surgery | 11 (10.7) |
| Arachnoid cyst surgery | 6 (5.8) |
| Spina bifida with myelomeningocele surgery | 1 (1.0) |
| **Clinical manifestation** |  |
| Fever | 97 (94.2) |
| Disturbance of consciousness | 57 (55.3) |
| Vomiting | 44 (42.7) |
| Headache | 12 (11.7) |
| Neck stiffness | 7 (6.8) |
| Convulsions | 8 (7.8) |
| Abdominal pain | 8 (7.8) |

**Supplementary Table 2. Distribution of Microorganisms Isolated from Cerebrospinal Fluids of Post-Neurosurgical Bacterial Meningitis Children.**

| **Characteristic** | **All Episodes**  **(n=103)** | **Survivors**  **(n=96)** | **Non-Survivors**  **(n=7)** |
| --- | --- | --- | --- |
| **Gram-Positive bacteria** |  |  |  |
| Staphylococcus epidermidis | 35 (34.0) | 35 (36.5) | 0 |
| Staphylococcus aureus | 7 (6.8) | 7 (7.3) | 0 |
| Methicillin-resistant Staphylococcus aureus | 3 (2.9) | 3 (3.1) | 0 |
| Staphylococcus haemolyticus | 5 (4.9) | 5 (5.2) | 0 |
| Enterococcus faecium | 3 (2.9) | 2 (2.1) | 1 (14.3) |
| Enterococcus faecalis | 1 (1.0) | 1 (1.0) | 0 |
| Other positive bacteria^*^ | 4 (3.9) | 4 (4.2) | 0 |
| **Gram-Negative bacteria** |  |  |  |
| Acinetobacter baumannii | 21 (20.4) | 18 (18.8) | 3 (42.9) |
| Klebsiella pneumoniae | 8 (7.8) | 7 (7.3) | 1 (14.3) |
| Pseudomonas aeruginosa | 5 (4.9) | 4 (4.2) | 1 (14.3) |
| Stenotrophomonas maltophilia | 3 (2.9) | 2 (2.1) | 1 (14.3) |
| Serratia marcescens | 3 (2.9) | 3 (3.1) | 0 |
| Escherichia coli | 2 (1.9) | 2 (2.1) | 0 |
| Other negative bacteria^**^ | 6 (5.8) | 6 (6.3) | 0 |
